# Properties of gold nanostructures sputtered on glass

**DOI:** 10.1186/1556-276X-6-96

**Published:** 2011-01-19

**Authors:** Jakub Siegel, Olexiy Lyutakov, Vladimír Rybka, Zdeňka Kolská, Václav Švorčík

**Affiliations:** 1 Department of Solid State Engineering, Institute of Chemical Technology, Technicka 5, 166 28 Prague, Czech Republic; 2 Department of Chemistry, J.E. Purkyně University, Ceské mládeze 8, 400 96 Usti nad Labem, Czech Republic

## Abstract

We studied the electrical and optical properties, density, and crystalline structure of Au nanostructures prepared by direct current sputtering on glass. We measured temperature dependence of sheet resistance and current-voltage characteristics and also performed scanning electron microscopy [SEM] analysis of gold nanolayers. It was shown that within the wide range of temperatures, gold nanolayers (<10 nm) exhibit both metal and semiconducting-like type of conductivity. UV/Vis analysis proved the semiconducting characteristic of intrinsic Au clusters. SEM analysis showed the initiatory stadium of gold layer formation to be running over isolated islands. Gold density calculated from the weight and effective thickness of the layers is an increasing function of the layer thickness up to approximately 100 nm. In thin layers deposited on solid surface, a lattice expansion is observed, which is manifested in the increase of the lattice parameter and the decrease of metal density. With increasing layer thickness, the lattice parameter and the density approach the bulk values.

## Introduction

Nanocrystalline thin solid films nowadays present enormous scientific interest, mainly due to their attractive novel properties for technological applications [1,2]. The most important prerequisite for the preparation of high-quality film is an understanding of its growth dynamics and structure in different phases of deposition.

In the course of the twentieth century, the theory of size-dependent effects in metal thin layers was further developed by numerous scientists, and various approaches to the problem were proposed. For isolated metal particles' behavior at exiguous dimensions (1D and 2D), quantum size effects are decisive, whereas for ultrathin metal layers both surface effects and quantum size effects must be considered [3,4]. These phenomena can be attributed to a high nanolayer and/or nanoparticle surface-to-bulk ratio. Hand in hand with the reduction of nanoparticle dimension, surface atoms' proportion increases dramatically; thus, commonly known physical properties of the bulk materials change, e.g., density and melting point of Au nanoparticle decreases [5-7]. Properties of metal layers are affected by electron scattering on phonons, on imperfections, and at layer boundaries. While the first two types of scattering occur also in bulk metal, the last one plays a role only in thin layers, and it is responsible for the reduction of the electric conductivity of thin layers [8]. Mathematical formula for the calculation of relaxation times for more than one scattering mechanism is given by Matthiessen's rule [8].

Gold is known as a shiny, yellow noble metal that does not tarnish, has a face-centered cubic structure, is non-magnetic, melts at 1,336 K, and has density a 19.320 g cm^-3^. However, a small sample of the same gold is quite different, providing it is tiny enough: 10-nm particles absorb green light and thus appear red. The melting temperature decreases dramatically as the sample size goes down [9]. Moreover, gold ceases to be noble, and 2- to 3-nm nanoparticles are excellent catalysts which also exhibit considerable magnetism [4,10]. At this size, Au nanoparticles also turn into insulators. Gold in the form of thin films is nowadays used in a vast range of applications such as microelectromechanical and nanoelectromechanical systems [11,12], sensors [13], electronic textiles [14], bioengineering [15], generator of nonlinear optical properties [16], or devices for surface-enhanced Raman scattering [17].

The optical and electrical properties of Au nanoparticles have been studied on samples prepared by atom sputtering deposition approach onto porous alumina in [18]. The electrical resistance measurement shows that the nanoparticles are conductive even at a small metal volume fraction. Due to the aggregation effect, the optical transmission spectra exhibited an enhanced transmition band around 500 nm arising from the surface plasmon resonance [18]. Many authors have developed theories of distortion of crystalline lattice in nanostructures, some of them being applicable on nanoparticles. Spherical nanoparticles surrounded 'by air' have different behaviors as nanostructures deposited on solid surface. While in spherical nanoparticles a dominant effect is a lattice compression [9,19-21], in other nanostructured materials (e.g., nanowires, nanolayers), a lattice expansion is observed [22,23]. The compression can be explained by the Young-Laplace equation for spherical particles and the effect of decreasing size and a curvature of surface. The expansion on the other hand can be due to imperfections of the lattice and the size surface effects on nanostructures. More important is the effect of lattice imperfections which, on the other hand, may lead to a density decrease.

In this work, we studied the electrical and optical properties, density, and crystalline structure of Au nanostructures prepared by sputtering on glass. Measurement of the sheet resistance of gold nanostructures at room and low (LN_2_) temperatures proved the metal or semiconductive-like characteristic of the structures. Scanning electron microscopy [SEM] analysis showed the gold layer growth to be running over isolated islands. The mechanism of charge transfer and the optical excitation of metal particles were determined by measuring the electrical sheet resistance and UV/Vis spectrometry, respectively. The UV/Vis spectra were interpreted in the frame of the well-known Tauc's model [24], and the optical band gap (*E*_g_^opt.^) of ultrathin Au structures was calculated as a function of structure thickness. X-ray diffraction [XRD] analysis provided information about the crystalline structure and the lattice parameter values. Density of Au was calculated from the weight (gravimetry) and the effective thickness of Au layers which were measured by atomic force microscopy [AFM].

### Experimental details

#### Substrate and Au deposition

The gold structures were sputtered on a 2 × 2-cm microscopic glass substrate, 1 mm thick, supplied by Glassbel Ltd., Czech Republic. Glass surface roughness of *R*_a _= 0.34 nm was measured at ""square 1.5 μm^2^. The sputtering was accomplished on a Balzers SCD 050 device from gold target (purity 99.99%, supplied by Goodfellow Ltd., Cambridge, UK). One slide was prepared during each sputtering operation. Deposition chamber was not equipped with a rotated sample holder. Under analogous experimental conditions, homogenous layers with uniform thickness were prepared [25]. The deposition conditions were the following: direct current Ar plasma, gas purity 99.995%, discharge power of 7.5 W, Ar flow approximately 0.3 l s^-1^, pressure of 5 Pa, electrode distance of 50 mm, electrode area of 48 cm^2^, and reaction chamber volume approximately 1,000 cm^3^. The sputtering times vary from 4 to 500 s.

#### Diagnostic techniques

Metal structure thickness for chosen sputtering times (effective thickness) was examined using AFM. The AFM images were taken under ambient conditions on a Digital Instruments CP II setup. The samples, 1 cm^2 ^in area, were mounted on stubs using a double-sided adhesive. A large area scanner was used, allowing an area up to 100 μm^2 ^to be imaged. A Veeco phosphorus-doped silicon probe CONT20A-CP with spring constant 0.9 N m^-1 ^was chosen. In the present experiment, structure homogeneity was tested by a scratch technique at ten different positions. The thickness of the structures was determined from the AFM scan done in contact mode [26]. Thickness variations do not exceed 5%. All scans were acquired at a scanning rate of 1 Hz.

The electrical properties of gold structures were examined by measuring the electrical sheet resistance (*R*_s_). *R*_s _was determined by a standard two-point technique using a KEITHLEY 487 picoampermeter. For this measurement, additional Au contacts, about 50 nm thick, were created by sputtering. The electrical measurements were performed at a pressure of about 10 Pa to minimize the influence of atmospheric humidity. The temperature dependence of *R*_s _was determined on the samples placed in a cryostat evacuated to the pressure of 10^-4 ^Pa. The samples were first cooled to the LN_2 _temperature and then gradually heated to room temperature. Typical error of the sheet resistance measurement did not exceed ± 5%.

The current-voltage [CV] characteristics were measured using picoampermeter KEITHLEY 487 (sheet resistance, >10^5 ^Ω) and multimeter UNI-T (sheet resistance, <10^5 ^Ω). The temperature dependence of CV characteristics was also determined. In that case, measured samples were placed into the cryostat at the temperature of liquid nitrogen and were gradually heated to room temperature.

XRD analysis was performed by an automatic powder refractometer Panalytical X'Pert PRO using a copper X-ray lamp (*λ*_CuKα1 _= 0.1540598 nm) equipped with an ultrafast semiconductor detector PIXcel. Measurement has passed on a symmetric Bragg-Brentano geometry. Diffractograms were registered in the angular range 2ϑ = (10° to 85°). Lattice parameter *a *of the cubic face-centered lattice of Au was calculated from diffraction lines location and its intensity using Rietveld's method. The lattice parameter could only be determined for samples with an Au thickness exceeding 10 nm.

UV/Vis spectra were measured using a Shimadzu 3600 UV-Vis-NIR spectrometer (Kyoto, Japan) in the spectral range from 200 to 2,700 nm. Evaluation of the optical spectra was performed using Film Wizard software with the aim of determining plasma frequency. Measured spectra were also interpreted in the frame of Tauc's model [24] using Tauc's equation *α*(*ν*) = *A*(*hν *- *E*_g_^opt^)^*x*^/*hν*, where *α *is the absorption coefficient of the substance, *E*_g_^opt ^is the substance optical band gap, *x *is the parameter that gives the type of electron transition, and factor *A *depends on the transition probability and can be assumed to be constant within the optical frequency range [26]. Optical band gap width, *E*_g_^opt^, of layers was assessed from the linear part of plot ((*α*(*ν*)⋅*hν*)^*x *^vs. *hν*). Indirect transition cannot be excluded in these layers, and therefore, *x *= 1/2 was used in the calculation.

Mettler Toledo UMX2 microbalance (Greifensee, Switzerland) was used for gravimetric determination of an amount of sputtered gold on a glass template. Density of Au layers was then calculated from the weight and effective layer thickness determined from the AFM scan.

Direct measurement of the layer thickness was accomplished by a SEM (JSM-7500F). The specimen for SEM examination was prepared by cross-sectioning of the metal-glass sandwich on a standard cross-section polisher, with focused ion beam (6-kV acceleration voltage).

## Results and discussion

### Thickness and morphology of Au structures

**Figure 1:**
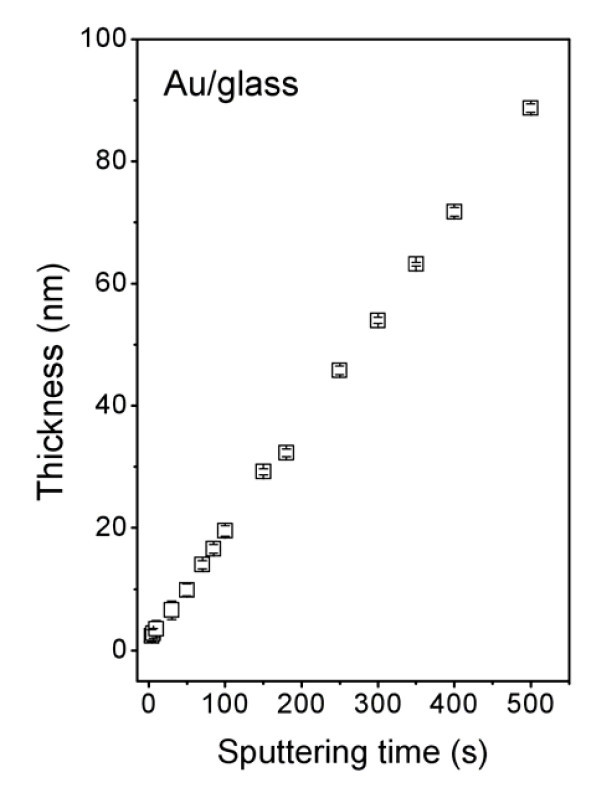
**Dependence of the gold structure thickness on sputtering time**.

Thickness of sputtered layers was measured by AFM. Thickness in the initiatory stadium of deposition (sputtering time, 50 s) was determined from the SEM image of the sample cross-section. Dependence of the layer thickness on sputtering time is displayed in Figure 1. Linear dependence between sputtering time and structure thickness is evident even in the initiatory stadium of the layer growth. This finding is in contradiction with results obtained earlier for Au sputtering on polyethyleneterephtalate [25]. In that case, the initiatory stadium of the layer growth was related to a lower deposition rate.

**Figure 2:**
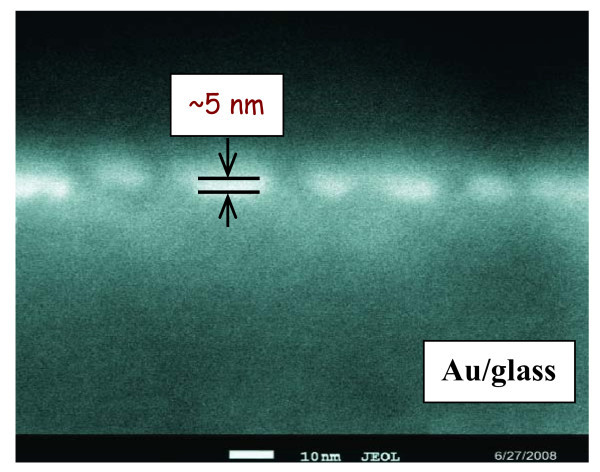
**SEM scan of the cut of gold structure on glass substrate**. Deposition time was 20 s. The cut was done with the FIB method.

In Figure 2, a SEM picture of the cross-section of the Au layer at its initiatory stadium of growth is shown. It is obvious that after approximately 20 s of Au deposition, flat, discrete Au islands (clusters) appear on the substrate surface. The flatness may indicate preferential growth of gold clusters in a lateral direction. When the surface coverage increases and the clusters get in close contact with each other, a coarsening sets in and becomes the dominant process. After the surface is fully covered, additional adsorption causes only the vertical layer growth, while the lateral growth is dominated by cluster boundary motion [27].

### Electrical properties of Au structures

**Figure 3:**
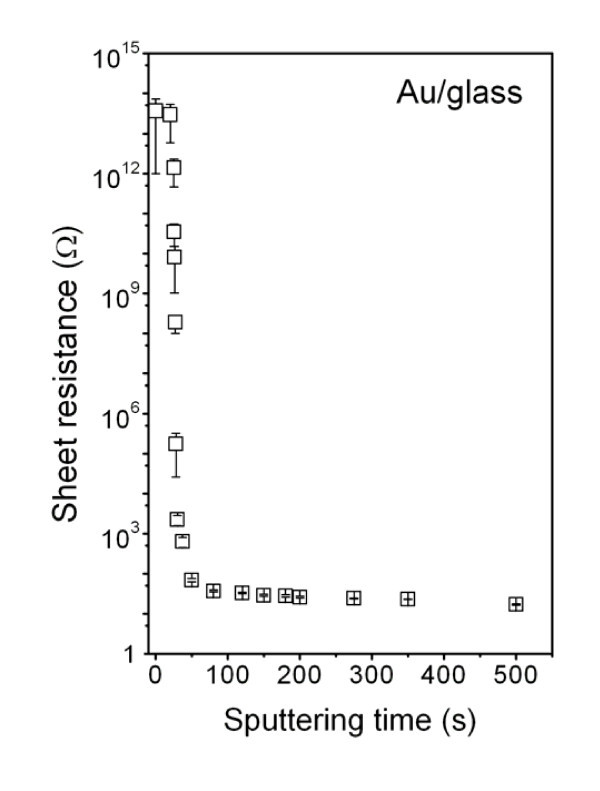
**Dependence of the sheet resistance of the gold structure on deposition time**.

Figure 3 shows the dependence of the sheet resistance of Au structure on the sputtering time. Precedence was given to the dependence on the sputtering time since the accuracy of AFM thickness determination is limited for short sputtering times. It is well known that a rapid decline of sheet resistance of the sputtered layer indicates a transition from the electrical discontinuous to the electrical continuous layer [28]. One can see that the most pronounced change in the sheet resistance occurs between 20 and 50 s of sputtering times, corresponding to the 5- to 10-nm range of the layer thickness. Thus, the layers with a thickness below 5 nm can be considered as discontinuous ones, while the layers with a thickness above 10 nm are definitely continuous. From the measured sheet resistance (Figure 3) and effective layer thickness, it is possible to calculate the layer resistivity *R *(Ω cm). One can see that the layer resistivities are about one order of magnitude higher than that reported for metallic bulk gold (*R*_Au _= 2.5 × 10^-6 ^Ω cm) [29]. The higher resistivity of thin gold layers is due to the size effect, in accord with the Matthiessen rule [8].

**Figure 4:**
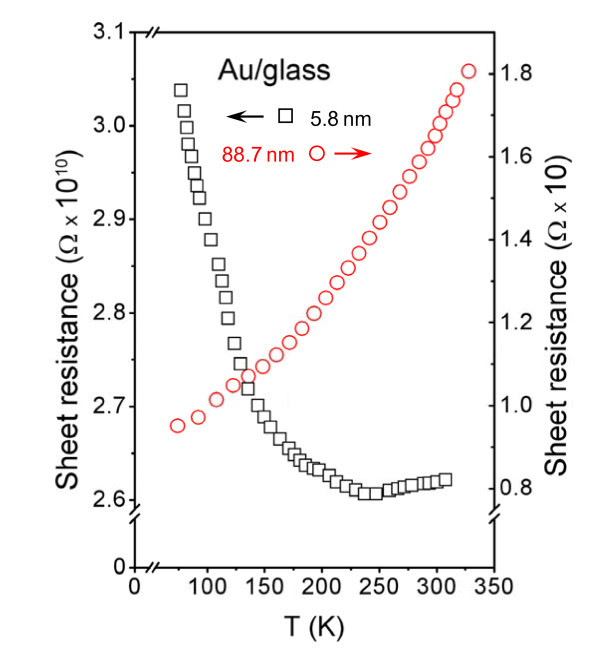
**Temperature dependence of the sheet resistance for two different structure thicknesses indicated in the figure**.

The temperature dependence of the sheet resistance for two particular structure thicknesses is displayed in Figure 4. One can see that the temperature dependence of the sheet resistance strongly depends on the structure thickness. For the layer about 89 nm thick, the resistance is an increasing function of the sample temperature, the behavior expected for metals. For the structure about 6 nm thick, the sheet resistance first decreases rapidly with increasing temperature, but above a temperature of about 250 K, a slight resistance increase is observed. The initial decrease and the final increase of the sheet resistance with increasing temperature are typical of semiconductors and metals, respectively. It has been referred elsewhere [4] that a small metal cluster can exhibit both metal and semiconductor characteristics just by varying the temperature. It is due to temperature-affected evolution of band gap and density of electron states in the systems containing low number of atoms. From the present experimental data, it may be concluded that for the thicknesses above 10 nm, the sputtered gold layers exhibit metal conductivity. In the thickness range from 5 to 10 nm, the semiconductor-like and metal conductivities are observed at low and high temperatures, respectively. Our further measurements showed that the layers thinner than 5 nm exhibit a semiconductive-like characteristic in the whole investigated temperature scale. Except for band gap evolution theory, typical semiconductor-like behavior may also originate from the tunneling effect of electrons through the discontinuous, separated Au clusters during electrical measurements. Since the probability of electron tunneling depends on the temperature, similarly, typical course of sheet resistance and, as will be shown later, CV characteristic may be affected right by this phenomenon.

**Figure 5:**
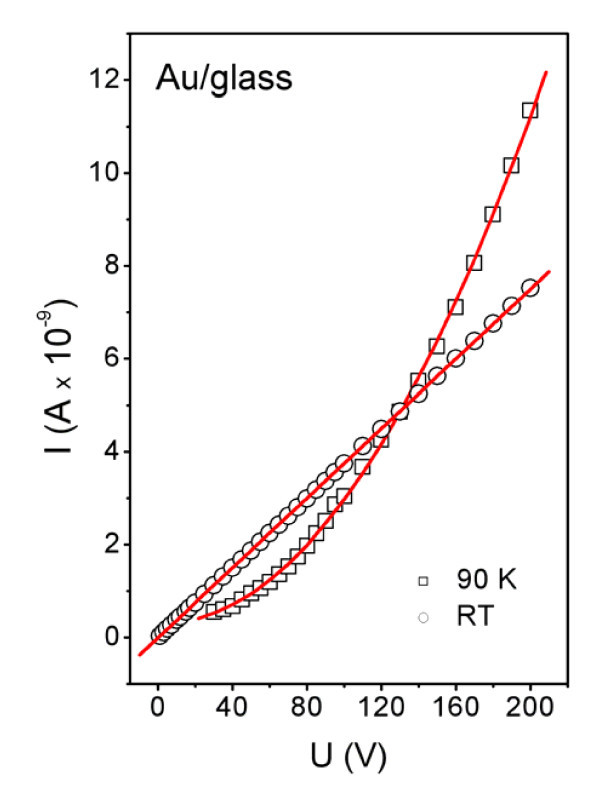
**Current-voltage characteristic of a 5.8-nm-thick Au structure measured at room temperature (*RT*) and at a temperature of 90 K**.

Figure 5 displays the CV characteristics of the 5.8-nm-thick Au layer measured at room temperature [RT] and at a temperature of 90 K (LN_2_). The CV curve at RT is strictly linear so that Ohm's law is valid and the layer exhibits metallic behavior. The CV curve obtained at 90 K grows exponentially so that it has a non-Ohmic characteristic typical of semiconductors. This is in a good accordance with the data of Figure 4 and the theory of band gap occurrence in metal nanostructures. While at RT the thermal excitation is big enough for electrons to overcome band gap, at 90 K, the band gap cannot be overcome. CV dependence measured at RT and 90 K on the 5.8-nm-thick Au layer confirmed former interpretation of the temperature dependence of the sheet resistence, i.e., metallic characteristic of the conductance at RT and the semiconductor one at low temperatures.

From the measurements of sheet resistance and CV characteristics result the semiconductor-like characteristic of Au at specific structure conditions (thickness, temperature). The observed semiconductor-like characteristic (decreasing resistance with increasing temperature, nonlinearity of CV characteristic) of ultrathin Au structures may originate from two undistinguishable phenomena. The first one results from a tunneling effect which occurs at discontinuous structures during resistance measurements [30]. The second one originates from the semiconductor characteristic of the intrinsic cluster itself, which occurs in metal nanostructures of sufficiently small proportions [4]. With respect to the experimental method used, it is impossible to distinguish which phenomenon prevails in prepared structures and contribute to the observed semiconductor-like behavior of Au nanostructures.

In order to investigate whether the intrinsic Au clusters forming ultrathin Au coverage exhibit semiconductor behavior, indeed we accomplished additional optical UV/Vis analysis.

### Optical properties of Au structures

**Figure 6:**
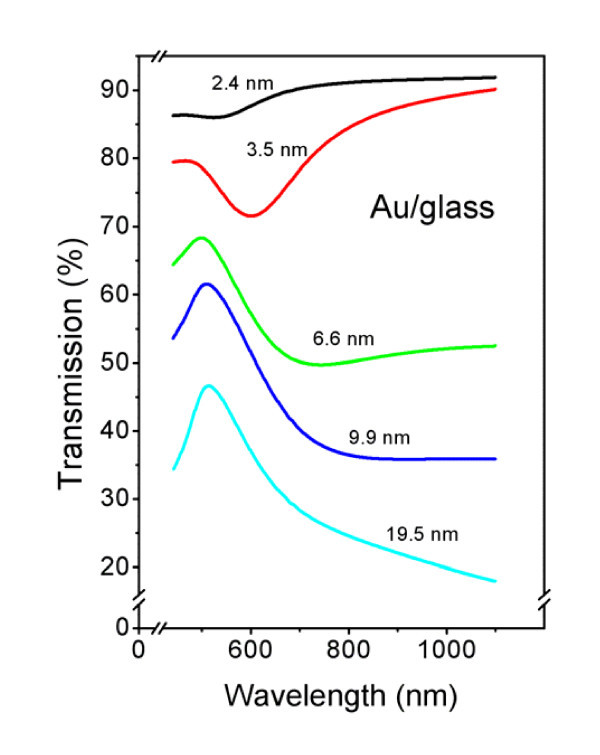
**Transmission spectra of gold layers for different structure thicknesses as indicated in the figure**.

Thin Au films exhibit structure-dependent UV/Vis optical spectra [28,31,32]. The localized absorption characteristic of Au films is highly sensitive to the surrounding medium, particle size, surface structure, and shape [33]. Transmission spectra from the samples with gold structures of various thicknesses are shown in Figure 6. Only the samples with the gold structure <20 nm thick, transmitting primary light beam enough, were examined. The spectra exhibit an absorption minimum around 500 nm which is slightly red-shifted with increasing film thickness. Pronounced absorption increasing at longer wavelength could be attributed to the surface plasmon resonance [34]. Discontinuous and inhomogeneous layers, with thickness ranging from 2.4 to 9.9 nm and composed of nanometer-sized metal clusters, exhibit absorption in the visible region attributed to the surface plasmon in the metal islands. The surface plasmon peak is shifted from 720 to 590 nm as the nominal layer thickness decreases from 19.5 to 2.4 nm. It is well known that optical absorption of island films of gold is a function of island density [35]. The absorption band resulting from bounded plasma resonance in the particles is shifted to longer wavelengths as the island density increases. As the thickness becomes greater, the absorption band is broadened due to a wider particle size distribution.

**Figure 7:**
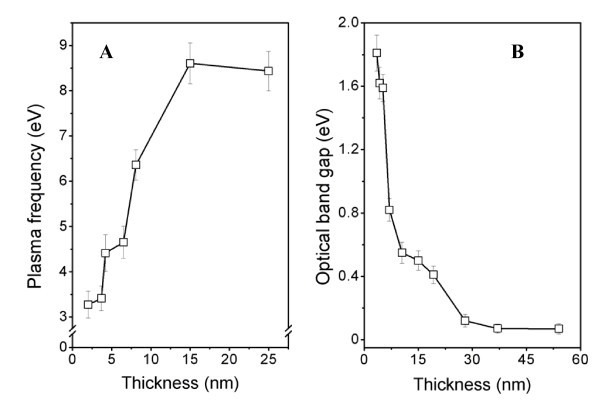
**Dependence of plasma frequency (A) and optical band gap (B) evaluated from the UV/Vis spectra on the thickness of deposited structures**.

Evaluation of the optical spectra was performed using Film Wizard software and a Maxwell-Garnett model was applied. In this model, Au films were described as a heterogeneous mixture of material and voids. With the aim of incorporating nanosize of gold clusters for the aforementioned material, the Lorentz-Drude behavior of the optical parameters was presumed. This approximation is a generalization of both the Lorentz oscillator and the Lorentz-Drude models and includes the effect of the free carrier contribution to the dielectric function and resonant transitions between allowed states. The best fits were obtained in the case of thickness from 2 to 15 nm. Main parameter of the chosen approximation, plasma frequency, is presented in Figure 7A as a function of the film thickness. As was predicted by the theory of Mie, the red shift [36] occurs with increasing cluster size (film thickness). Additionally, it is evident that plasma frequency strongly depends on the film thickness. The plasma frequency increases with increasing layer thickness, and for thicknesses above 15 nm, it reaches typical 'bulk' value of gold, 9.02 eV. It is well known that the plasma frequency is closely related to the concentration of the free carrier [37]. From Figure 5, it can be concluded that the concentration of free carriers is an increasing function of the film thickness. This result is in good agreement with previous studies [30]. Increase of free carrier concentration with increasing nanostructure thickness is a direct evidence of the tunneling effect of electrons between isolated gold clusters [30].

The UV/Vis spectra were also interpreted in the frame of Tauc's model [24] (see also above) and the optical band gap (*E*_g_^opt.^) calculated as a function of the structure thickness. The *E*_g_^opt. ^as a function of the structure thickness is shown in Figure 7B. A non-zero value of *E*_g_^opt. ^was detected in the case of Au structure thicknesses ranging from 2 to 30 nm, which corresponds with the sputtering times between 4 and 150 s. Apart from electrical measurements, optical methods do not require any conductive path between separated clusters during measurement. That is why optical-based methods are able to separate the contribution of tunneling effects to the properties of Au nanostructures, which cannot be omitted during electrical measurements of discontinuous metal layers. Optically analyzed evolution of band gap thus unambiguously confirms the semiconductive characteristic of intrinsic clusters forming Au nanolayers. However, even after the electrically continuous layer is formed (sputtering time of approximately 50 s, which corresponds to a structure thickness of approximately 10 nm), which is characterized by the creation of a conductive path between isolated clusters and a rapid decline of sheet resistance (see Figures 1 and 3), there still must exist regions of separated Au clusters in deposited layer which contribute to non-zero *E*_g_^opt. ^up to the structure thickness of approximately 30 nm (see Figure 7B).

### Lattice parameter and density of Au structures

**Figure 8:**
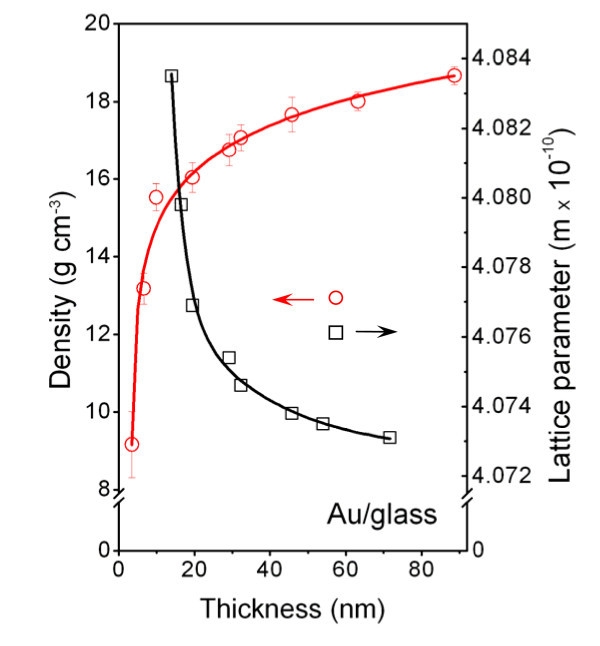
**Dependence of lattice parameter (*square*) and density (*circle*) on Au layer thickness for glass substrate**. The density was calculated from Au layer effective thickness and mass.

It has been published elsewhere [5,38] that the lattice parameter of metals prepared in the form of a thin layer by a physical deposition is not a material constant but depends strongly on the layer thickness. Figure 8 displays the dependence of the Au lattice parameter on layer thickness derived from the present XRD measurements. The dependence exhibits a monotonous decline of the lattice parameter with increasing layer thickness. This can be explained by the internal stress relaxation during the growth of gold clusters (see Figure 2 and [39]).

With the aim of finding how the decline of lattice parameter influences the density of gold structures, we measured the effective thickness and the mass of deposited structures and calculated the effective density in a standard way. In Figure 8, the dependence of the density on the layer thickness is shown. The density increases with increasing layer thickness, and for about a 90-nm-thick layer, it achieves the density of bulk gold. The reduced density of thinner structures is probably due to the higher fraction of free volume in gold nanoclusters. As the gold clusters become greater [27], the free volume fraction decreases and the gold density gradually increases. It was reported earlier [40] that gold layers with thicknesses above 100 nm prepared on glass substrate exhibit quite a uniform density, with a mean value of 19.3 g cm^-3 ^typical of bulk material. Theoretical Au density was calculated from the value of lattice parameter [41].

## Conclusions

We observe a linear dependence between the sputtering time and the structure thickness even in the initial stadium of the Au growth. After the stage of nucleation, the growth of Au clusters proceeds mainly in the lateral direction. A rapid decline of the sheet resistance of the gold layer with increasing structure thickness indicates a transition from the discontinuous to the continuous gold layer. From the dependence of the sheet resistance on the sample temperature and from the measured CV characteristics of Au structures, it follows that the gold layers thicker than 10 nm exhibit a metallic characteristic. Structures with thicknesses between 5 and 10 nm exhibit a semiconductor-like characteristic at low temperatures and metalloid conductivity at higher temperatures. Layers with thicknesses below 5 nm exhibit semiconductive-like properties in the whole investigated temperature range. Optical absorption of the structures at the initial phase of the layer growth is a function of the gold cluster density. Plasma frequency (concentration of free carrier) increases with the layer thickness. UV/Vis analysis proved the semiconducting characteristic of intrinsic Au clusters. XRD measurements proved the monotonous decline of the lattice parameter with increasing structure thickness. Measurements of the effective thickness and weight of deposited structures showed that the Au density is an increasing function of structure thickness. For the layer thicknesses above 90 nm, the layer density achieves the bulk value.

## Competing interests

The authors declare that they have no competing interests.

## Authors' contributions

JS carried out thickness and resistance measurements at RT, participated in Au density determination. He designed and drafted the study. OL carried out resistance measurements at low temperature and optics measurements together with its evaluation. VR participated in the evaluation of optical spectra and electrical measurements. ZK carried out the Au density and lattice parrameter. VS concieved of the study and participated in its coordination.
